# Antigen-specific immune reactions to ischemic stroke

**DOI:** 10.3389/fncel.2014.00278

**Published:** 2014-09-12

**Authors:** Xabier Urra, Francesc Miró, Angel Chamorro, Anna M. Planas

**Affiliations:** ^1^Functional Unit of Cerebrovascular Diseases, Hospital ClínicBarcelona, Spain; ^2^August Pi i Sunyer Biomedical Research Institute (IDIBAPS)Barcelona, Spain; ^3^Department of Brain Ischemia and Neurodegeneration, Instituto de Investigaciones Biomédicas de Barcelona (IIBB), Consejo Superior de Investigaciones Científicas (CSIC)Barcelona, Spain

**Keywords:** stroke, antigens, autoimmunity, tolerance, brain, lymphoid tissue

## Abstract

Brain proteins are detected in the cerebrospinal fluid (CSF) and blood of stroke patients and their concentration is related to the extent of brain damage. Antibodies against brain antigens develop after stroke, suggesting a humoral immune response to the brain injury. Furthermore, induced immune tolerance is beneficial in animal models of cerebral ischemia. The presence of circulating T cells sensitized against brain antigens, and antigen presenting cells (APCs) carrying brain antigens in draining lymphoid tissue of stroke patients support the notion that stroke might induce antigen-specific immune responses. After stroke, brain proteins that are normally hidden from the periphery, inflammatory mediators, and danger signals can exit the brain through several efflux routes. They can reach the blood after leaking out of the damaged blood-brain barrier (BBB) or following the drainage of interstitial fluid to the dural venous sinus, or reach the cervical lymph nodes through the nasal lymphatics following CSF drainage along the arachnoid sheaths of nerves across the nasal submucosa. The route and mode of access of brain antigens to lymphoid tissue could influence the type of response. Central and peripheral tolerance prevents autoimmunity, but the actual mechanisms of tolerance to brain antigens released into the periphery in the presence of inflammation, danger signals, and APCs, are not fully characterized. Stroke does not systematically trigger autoimmunity, but under certain circumstances, such as pronounced systemic inflammation or infection, autoreactive T cells could escape the tolerance controls. Further investigation is needed to elucidate whether antigen-specific immune events could underlie neurological complications impairing recovery from stroke.

## Introduction

Ischemic stroke induces acute brain damage and cell death. The lack of sufficient energy to maintain the membrane potential of the cells causes necrosis. Necrosis, in contrast to apoptosis or other forms of cell death, promotes a strong inflammatory response after the intracellular content spills into the extracellular environment. Stroke fuels a sterile local and systemic inflammatory response with the release of danger signals or damage-associated molecular patterns from the injured tissue (Iadecola and Anrather, 2011), which could in turn stimulate a pro-thrombotic cascade, as well as activate the innate and adaptive arms of the immune system, with still poorly understood consequences. Inflammation is necessary to clear the dead cells and cell debris but it needs to be tightly regulated to avoid excessive release of neurotoxic mediators, damage to the blood-brain barrier (BBB), and cause uncontrolled activation of the immune system. Cytokines, chemokines and adhesion molecules participate in the recruitment of peripheral leukocytes that are attracted to the injury site (Gelderblom et al., 2009). Extracellular proteolytic enzymes are rapidly activated degrading the extracellular matrix and activating pro-zymogens that cleave proteins, all in preparation for further tissue remodeling (Yang et al., 2013). These proteolytic processes can expose otherwise hidden epitopes that can act as danger signals, release pro-inflammatory mediators, and damage the BBB. Inflammatory mediators cause indirect activation of antigen-presenting cells (APCs) driving T cell proliferation and clonal expansion, but indirectly activated APCs cannot support differentiation of CD4+ T cells into Th1 effectors *in vivo* (Spörri and Reis e Sousa, 2005). Therefore, inflammation allows APCs to sense danger but direct danger signal recognition is necessary to better identify the quality of the danger (Spörri and Reis e Sousa, 2005). Danger signals activate pattern-recognition receptors, such as toll-like receptors (TLR), inducing full maturation of APCs (Janeway and Medzhitov, 1999). Although microglia and macrophages can present antigen, dendritic cells (DCs) are the professional APCs, and they are found in the brain after stroke (Felger et al., 2010). Dendritic cells capture antigen mainly in its immature stage and then undergo maturation enabling them to efficiently present antigen by increasing the expression of MHC II and co-stimulatory molecules, and producing cytokines that stimulate T cells (Steinman and Nussenzweig, 2002). For efficient naïve T cell stimulation, peripheral antigen-loaded DCs migrate toward tissue-draining lymph nodes. However, it is currently unknown whether stroke-induced brain DCs migrate to the cervical lymph nodes due to the absence of direct lymphatic connection. Also, soluble proteins and protein fragments normally confined to brain cells or trapped in the extracellular matrix could reach the periphery through the leaky BBB or through the drainage pathways of interstitial fluid and cerebrospinal fluid (CSF; Cserr et al., 1992; Weller et al., 2009; Carare et al., 2014), as we will describe below. Once in the periphery, brain proteins could be taken up by APCs, processed, and presented to T cells in lymphoid tissue.

Brain antigens were found in draining lymphoid tissue of stroke patients (Planas et al., 2012) and mice (van Zwam et al., 2009), suggesting that antigen-specific immune reactions could take place after stroke. This possibility does not necessarily imply that an immune attack to the brain would be expected after stroke. There are indeed powerful mechanisms to control autoimmunity ensuring tolerance (Hogquist et al., 2005), and regulatory mechanisms operate in stroke (Liesz et al., 2009). However, tolerance can be breached under certain circumstances and several lines of evidence support that inflammation and/or infection can facilitate autoimmune reactions in experimental animal models of brain ischemia (Becker et al., 2005; Gee et al., 2009; Zierath et al., 2010) and in human stroke (Becker et al., 2011). While this concept is not new and some previous reviews have addressed related issues (e.g., Becker, 2009, 2012; Vogelgesang and Dressel, 2011; Vogelgesang et al., 2014), we will discuss the phenomenon of induction of tolerance in experimental stroke, the presence of autoantibodies in stroke patients, the presence of antigen-specific T cells in stroked animals and humans, in the context of antigen presentation, and we will address the possible relevance of such phenomena in medium or long-term stroke outcome.

The effects we will be referring to in this manuscript involve an adaptive immune response that is different from the damaging effects of T cells found in the very acute phase of stroke. Acute deleterious effects of T cells were perceived through the protection detected in lymphocyte-deficient mice after cerebral ischemia/reperfusion, and the capacity to reverse this phenomenon by adoptive transfer of T cells (Yilmaz et al., 2006; Hurn et al., 2007; Kleinschnitz et al., 2010). The acutely detrimental T-cell-mediated actions are mediated, at least in part, by impairment of the brain microcirculation through leukocyte adhesion to brain vessel walls (Yilmaz et al., 2006) promoting secondary microthrombosis (Kleinschnitz et al., 2010, 2013). Early detrimental effects of innate natural killer (NK) lymphocytes have also been reported in brain ischemia (Gan et al., 2014). These very acute lymphocyte effects contribute to the innate immune response to stroke (Magnus et al., 2012) but are not antigen specific and will not be addressed in this review.

## Brain protein release to the periphery in ischemic stroke

After cerebral ischemia, metabolites of the ischemic molecular cascade and CNS proteins are released to the periphery, putatively enabling the generation of autoimmune responses against brain-specific antigens (Iadecola and Anrather, 2011; Chamorro et al., 2012). Protein markers of cerebral damage, including myelin basic protein (MBP), neuron-specific enolase (NSE), S100β, and glial fibrillary acidic protein (GFAP), are found in CSF and serum after stroke. Moreover, the concentration of these proteins is related to the severity of the neurological deficits (Jauch et al., 2006) and the extent of the brain lesion on neuroimaging in humans (Jauch et al., 2006) and experimental animals (Gelderblom et al., 2013). High levels of MBP and S100β are also predictive of poor functional recovery (Strand et al., 1984; Missler et al., 1997; Herrmann et al., 2000; Jauch et al., 2006). Brain antigens are not only found in the CSF and serum but also in lymphoid tissue of stroke patients (Planas et al., 2012; Gómez-Choco et al., 2014) where they can be presented by APCs and could trigger autoimmune or tolerogenic immune responses.

## Autoantibodies

The presence of IgG immunoglobulin bands in the CSF of stroke patients was reported a long time ago (Roström and Link, 1981), suggesting that the release of brain antigens could be followed by intrathecal B-cell responses. Other researchers have confirmed the presence of specific IgG, IgM and IgA autoantibodies in the CSF (Prüss et al., 2012), and this is often accompanied by pleocytosis and altered albumin quotients of CSF/serum indicating BBB dysfunction. Overall, these findings suggest local activation of the immune system and possibly a pathogenic role of specific autoantibodies in stroke patients. Autoantibodies were also reported in serum. For example, anti-neurofilament antibodies were elevated after stroke, while antibodies against a ubiquitous antigen, cardiolipin, did not increase, again suggesting that brain antigens exposed in stroke are able to initiate an specific antibody response (Bornstein et al., 2001). Antibodies against the NR2A/2B subtype of N-methyl-D-aspartate (NMDA) receptor in serum are also more frequent in patients with transient ischemic attack (TIA) and acute ischemic stroke compared to non-stroke patients (patients admitted with suspected stroke but who had a non-stroke diagnosis at discharge) or healthy controls (Weissman et al., 2011), with high sensitivity, specificity, and predictive values (Dambinova et al., 2003). The presence of these autoantibodies may harbinger an increased risk of stroke as identification of anti-NMDA antibodies in patients before cardiopulmonary bypass surgery was associated with the development of neurological deficits and stroke (Bokesch et al., 2006). Patients with TIA and with ischemic stroke had similar titers of antibodies to NR2A/2B, suggesting that minor ischemic insults, and even subclinical lesions, may be sufficient to activate immunity (Dambinova et al., 2003). It is possible that the loss of BBB integrity is critical to allow autoantibodies to exert pathological effects, since to some extent the presence of autoantibodies is also seen in healthy subjects (Hammer et al., 2013).

Antibody-producing B cells, although not numerous in lesions, contribute to anti-atherosclerotic activity, perhaps as a result of specific antibodies against plaque antigens, binding of antibodies to inhibitory Fc receptors, or cytokines produced by B cells. Spleen B cells are particularly effective inhibitors of atherosclerosis (Caligiuri et al., 2002), possibly because certain natural antibodies produced by some of these cells recognize phosphorylcholine, a molecule present in oxidized LDL, apoptotic cell membranes, and the cell wall of *Streptococcus pneumoniae*. These antibodies may contribute to the elimination of oxidized LDL and dead cells, as well as to the defense against pneumococcal infections. Interestingly, persons who have undergone splenectomy have increased susceptibility not only to pneumococcal infections but also to coronary artery diseases (Sherer and Shoenfeld, 2006). However in experimental animals, intrastriatal, but not systemic, administration of splenic CD19+ B-cells reduced infarct volume in B-cell deficient mice (Chen et al., 2012) suggesting that B-cells exert protective effects against ischemic brain injury. In human studies we found a positive association between the number of circulating CD19+ B cells and good functional outcome after stroke (Urra et al., 2009a). Therefore, the role of B cell responses and antibody production in stroke outcome is still far from being fully understood. Whether circulating autoantibodies impair long-term functional outcome after stroke has not been demonstrated so far but it is plausible that they could exert pathogenic effects if they reached the brain under particular situations where the functionality of BBB was perturbed.

## Anatomic paths that brain proteins can follow to reach the periphery

**Figure 1:**
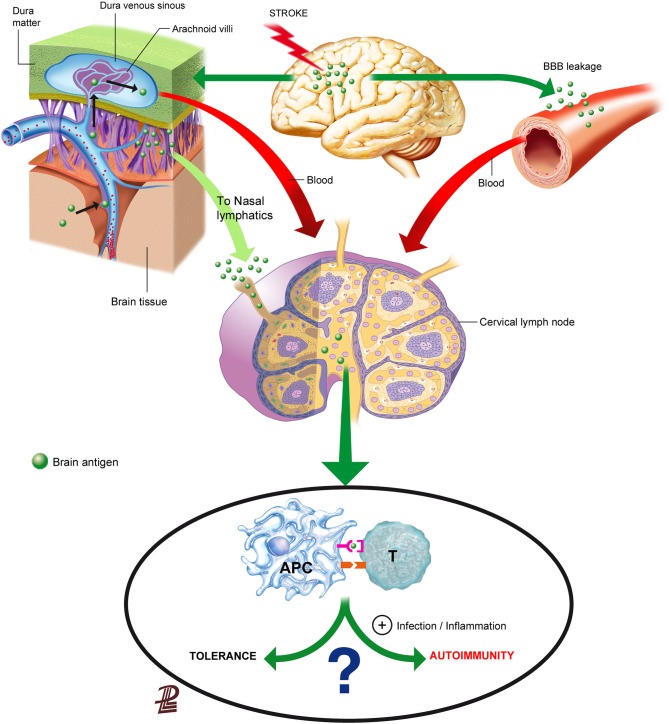
**Routes for the presentation of brain antigens in the lymphoid tissue**. Brain antigens can reach regional lymphoid tissue through various routes after stroke. Ischemic stroke increases BBB permeability that can allow the leakage of proteins or peptides into the blood. Soluble proteins/peptides in brain interstitial fluid can reach the CSF through perivascular spaces and flow to the venous blood through the arachnoid villi or drain from the CSF may also drain along the arachnoid sheaths of the olfactory nerves towards the regional lymphatics. Although brain antigens are depicted as traveling in a soluble form in the figure, it is also possible that peptides were internalized, processed and presented by APCs in the brain, and that they reached regional lymph nodes through migrating APCs. Antigen presentation in the lymph nodes will usually induce tolerance to self-antigens, but under some circumstances that favor an inflammatory milieu presentation of brain antigens may result in activation of autorreactive T cells.

Brain components could reach the lymphoid tissue either through the blood after crossing the leaky BBB, through interstitial fluid drainage to the blood or to the cervical lymph nodes, or after their local capture by migrating APCs (Figure 1). Understanding the route of access of brain proteins to the lymphoid tissue is not solely an anatomic issue, because depending on the way and form of entry to the lymphoid tissue, brain proteins/peptides will be encountered by different immune cells in different environments that may strongly influence the subsequent type of response, as we will discuss below.

Increased BBB permeability is a characteristic of stroke that could facilitate the leakage of brain proteins or protein fragments to the bloodstream. Proteolytic enzymes activated after stroke damage structural BBB components and cause BBB breakdown (Yang and Rosenberg, 2011). Neutrophils contain high levels of metalloproteinases (MMPs), such as MMP-9, and other destructive proteolytic enzymes, normally prepared to fight microbes, and contribute to the proteolytic activity after brain ischemia (Justicia et al., 2003; Gidday et al., 2005). Exposure of the neurovascular unit to such proteolytic activity cleaves tight junction proteins and damages the basement membranes, eventually causing BBB breakdown in acute stroke (Cunningham et al., 2005; Ludewig et al., 2013; Yang et al., 2013). However, BBB dysfunction may involve different degrees of cellular, structural, and molecular changes ranging from transient reversible dysfunction to more long-lasting alterations. Results obtained with different methods assessing BBB permeability support that there must be different grades of BBB dysfunction after brain ischemia (Nagaraja et al., 2008). The extent and nature of such alterations might exert some selectivity in the actual leakage of CNS protein components to the circulation, based on their size and other physical or biochemical features.

Besides exiting the brain through the leaky BBB, brain molecules can reach the periphery through the anatomic paths that allow for direct interstitial fluid drainage by bulk flow to the blood or to the lymphatics (Cserr et al., 1992; Weller et al., 1992; Abbott, 2004). The physical connection circuitry out of the brain towards the immune system enables draining of CSF into the lymphatics (Cserr et al., 1992; Weller et al., 1992). Interstitial extracellular fluid from the brain tissue drains to the CSF through perivascular spaces surrounding brain arterioles, but not venules (Arbel-Ornath et al., 2013; Carare et al., 2014). Perivascular spaces are connected to the subarachnoid space, allowing for fluid drainage to the venous blood through the arachnoid villi located at the dural venous sinuses (Cserr et al., 1992; Ransohoff et al., 2003). In addition, fluid from the subarachnoid space drains directionally to the cervical lymph nodes (Cserr et al., 1992; Zhang et al., 1992; Carare et al., 2014). Olfactory nerves are ensheated by arachnoid membranes allowing the drainage of CSF to the nasal mucosa through the cribiform plate, reaching nasal lymphatics, and from there, the CSF drains to the cervical lymph nodes (Harling-Berg et al., 1989; Cserr et al., 1992). An example of the functional relevance of this pathway is that the cervical lymph nodes are involved in the systemic humoral immune response to antigen infused into rat cerebrospinal fluid (Harling-Berg et al., 1989). Impairment of drainage of interstitial fluid out of the brain is believed to play a crucial role in the failure to adequately eliminate amyloid-β from the brain promoting its accumulation in the arterial walls in the elderly, and more prominently in patients with cerebral amyloid angiopathy (Weller et al., 2008; Hawkes et al., 2011, 2014; Arbel-Ornath et al., 2013). Since the force driving perivascular drainage is attributed to arterial vessel pulsations, it is not surprising that fluid drainage was found to be obstructed in an experimental model of focal brain ischemia induced by photothrombosis (Arbel-Ornath et al., 2013). This finding implies that stroke could impair the possible transfer of brain antigens from the interstitial fluid of the ischemic tissue to the CSF, but might not necessarily prevent the transfer connection from CSF to the cervical lymph nodes. In any case, ischemia-induced bulk-flow alterations might reverse, at least in part, at reperfusion.

Besides the possible exit of brain antigen from the brain tissue in a soluble form through the pathways indicated above (Figure 1), antigen can also be taken up locally in the brain by APCs. Dendritic cells (CD11c+) expressing MHC II and co-stimulatory molecules are found in the ischemic tissue (Felger et al., 2010) suggesting that they can present antigen. Migrating DCs traffic from peripheral tissues to their nearest lymph nodes through a process orchestrated by CCR7 in response to chemokines CCL19 and CCL20 (Förster et al., 1999), but other pathways could also be implicated such as sphingosine-1-phosphate (S1P) signaling (Czeloth et al., 2005) or the MHC II invariant chain (CD74) (Faure-André et al., 2008). Since the brain lacks lymphatic vessels, it is currently unknown whether mature DCs carrying antigen can migrate from the injured brain tissue to the peripheral lymphoid tissue. It was reported that cells from the brain could reach the deep cervical lymph nodes through the nasal submucosa (Cserr et al., 1992), supporting that cells in the subarachnoid space might be able to reach the draining lymph nodes. This possibility would imply that APCs could follow chemoattractant gradients along the anatomic connections between the CSF and the cervical lymph nodes playing a natural role in the process of brain immunosurveillance. A study injecting DCs into the brain parenchyma showed little migration from their site of injection and cells did not reach the cervical lymph nodes, while intra-CSF-injected DCs did, and they preferentially targeted B-cell follicles rather than T-cell-rich areas suggesting that they favored humoral responses rather than cellular immunity (Hatterer et al., 2006). Efflux of solutes injected into the interstitial fluid of the brain was found to take place along basement membranes in the walls of capillaries and arteries (Carare et al., 2008). These basement membranes are very narrow, about 100 nm-thick, and therefore in normal conditions this pathway does not seem to be large enough to allow the trafficking of cells (Carare et al., 2008, 2014). Therefore, further studies are needed to find out whether and how mature APCs exit the brain to reach the peripheral lymphoid tissue after stroke.

## Antigen presentation: immunity vs. tolerance

We identified in human stroke an increased presence of brain-derived antigens in migrating DCs and macrophages in lymphoid tissue located within the draining pathways of the CNS (Planas et al., 2012). This finding extends previous observations accrued in the cervical lymph nodes of rodents with ischemic brain damage or autoimmune disease, and in patients with multiple sclerosis (de Vos et al., 2002). These studies raise the issue of whether APCs in lymph nodes can present brain antigens to T cells after acute brain damage, and whether they can induce immune reactions that will either exacerbate the brain injury or promote mechanisms of T-cell tolerance. Central tolerance ensures, through negative selection, the elimination of most T cells recognizing self-antigens in the thymus. This process is complemented with peripheral tolerance that guarantees tolerization of autoreactive T cells and involves the action of peripheral APCs (Steinman and Nussenzweig, 2002). Cytoplasmic endogenous peptides are processed mostly through the proteasome, loaded into MHC class I in the endoplasmic reticulum, and shuttled to the cell membrane through the secretory pathway for presentation by MHC I in all cells, allowing recognition by CD8+ cytotoxic lymphocytes (Hulpke and Tampé, 2013). In contrast, exogenous peptides are presented through MHC class II after capture by APCs through endocytosis, including pinocytosis, phagocytosis, and receptor-mediated endocytosis (Wilson and Villadangos, 2005). Antigen presentation through MHC II elicits responses in CD4+ T helper (Th) cells. The exception to this role is cross-presentation of exogenous antigens by APCs through MHC I. This process occurs when exogenous peptides from the cell environment reach the cytoplasm, are presented through MHC I, and activate CD8+ T cells (Heath and Carbone, 2001; Joffre et al., 2012).

Presentation of exogenous proteins by APCs through MHC II, and also through cross-presentation, is essential for T cell priming against invading pathogens and for the induction of tolerance to self-tissue-specific proteins. Immune tolerance to self-antigens is based on the regulation of autoreactive lymphocytes by several mechanisms including deletion, clonal anergy, or suppression by regulatory T cells (Tregs) and other regulatory cells (Goodnow et al., 2005), and is dependent on the features of the interactions between T cells and APCs (Heath and Carbone, 2001). Dendritic cells are tolerogenic according to their maturation and functional status and are able to delete or silence autoreactive T cells and facilitate the development of Tregs (Rescigno, 2010; Ganguly et al., 2013), which play critical roles in controlling autoimmunity (Sakaguchi et al., 2001). Endogenous Tregs increase after experimental brain ischemia (Offner et al., 2006), and they proliferate and accumulate in the ischemic tissue up to 30 days after middle cerebral artery occlusion (MCAO; Stubbe et al., 2013). Regulatory T cells are involved in suppressing potentially harmful immune responses in stroke through the production of interleukin 10 (IL-10; Liesz et al., 2009), although some studies found no differences in the neurological outcome of stroke after depleting CD25(+) Tregs (Stubbe et al., 2013). Enhancing the immunosuppressive function of Tregs with histone deacetylase inhibitors was reported to reduce ischemic brain damage (Liesz et al., 2013). Immunotherapies with Tregs are currently under investigation to promote immune tolerance in various diseases (Singer et al., 2014). However, the effects of exogenous Treg administration in experimental brain ischemia are controversial, with some studies reporting protective actions (Li et al., 2013a,b), and other studies finding damaging effects related to the non-antigen specific impairment of the microcirculation (Kleinschnitz et al., 2013), as attributed to other T cells (Kleinschnitz et al., 2010). Regulatory B cells (Bregs) also produce IL-10 and TGF-β and exert immunomodulatory functions contributing to the maintenance of self-tolerance (Vadasz et al., 2013). Interleukin 10 producing Bregs were found to exert protection in experimental brain ischemia in mice, and Breg administration increased Treg numbers and the expression of the co-inhibitory receptor programmed death (PD)-1 (CD279) (Ren et al., 2011a; Bodhankar et al., 2013a).

The pathway of antigen access to the draining lymphoid tissue might influence the type of immune response since immature DCs that are resident in the lymph nodes efficiently take up, process and present antigen to induce tolerance (Inaba et al., 1997). Soluble brain peptides traveling through afferent lymphatic vessels to lymph nodes could be taken up by APCs or could be degraded extracellularly since immature DCs secrete proteases able to generate antigens that are eventually loaded on surface MHC II molecules (Santambrogio et al., 1999). Therefore, brain proteins or peptides reaching the lymphoid tissue in a soluble form could be internalized by resident macrophages or by immature DCs following prior extracellular degradation. We did not detect brain-derived antigens in lymphoid tissue resident DCs after stroke, but the brain-antigen loaded APCs were compatible with macrophages and migratory DCs (Planas et al., 2012). Antigen presentation by macrophages or DCs seems to be dependent on the form of antigen delivery to the cells, possibly due to different cell-type dependent internalization mechanisms. Dendritic cells preferentially internalize protein fragments whereas native proteins are better taken up by macrophages after receptor-mediated internalization or phagocytosis of apoptotic cells (Tsark et al., 2002). Dendritic cells are more efficient APCs than macrophages since they process antigen through a mechanism better preserving epitopes for T cell activation (Savina et al., 2006), whereas macrophages induce a very strong lysosomal acidification for protein degradation and display a different repertoire of lysosome proteases than DCs (Burster et al., 2005). Interestingly, we observed contacts between brain-antigen immunoreactive APCs and lymphoid resident DCs (Planas et al., 2012), which suggested the possibility of cargo exchange between these cells. It has been reported that antigens can be transferred from migrating APCs to lymph node-resident DCs for presentation and priming of cytotoxic lymphocytes (Allan et al., 2006). Cell-to-cell antigen transfer can be mediated through gap junctions (Neijssen et al., 2005). A recent study showed connexin 43 gap junction-mediated transfer of antigen from macrophages to CD103+ DCs, and the involvement of this process in the establishment of oral tolerance (Mazzini et al., 2014). Another pathway of possible antigen transfer between cells is via exosomes, i.e., externalized endosomal vesicles secreted by different cell types including DC and B cells. Exosomes are formed by direct fusion of membranes of the MHC II-enriched compartment with plasma membrane containing co-stimulatory molecules such as CD86 (Raposo et al., 1996; Denzer et al., 2000; Harvey et al., 2007). These vesicles are transferred from cell-to-cell by adherence to the cell surface rather than by membrane fusion (Denzer et al., 2000). The expected response to antigen transfer mediated by gap junctions or by exosomes would be different since the latter carry co-stimulatory molecules. It is unknown whether any of these mechanisms could support the transfer of brain antigen from macrophages or migrating DCs to resident DCs in the lymph node after stroke, and whether such processes could be involved in tolerization.

The maintenance of peripheral tolerance involves cross-presentation (Belz et al., 2002). CD8+ T cells recognizing self-antigen with high affinity are eliminated in the peripheral lymph nodes, and this process is termed cross-tolerance (Redmond and Sherman, 2005). Myelin-specific CD8+-T-cells play a pathogenic role in experimental models of multiple sclerosis (Huseby et al., 2001). Cross-presentation requires that the internalized Ag in the endosomal compartment access the cytosol. This process is regulated in diverse ways by chaperone proteins of the heat-shock family (HSPs) that prevent protein aggregation and misfolding (Srivastava, 2002). Heat-shock proteins mediate the transfer of antigenic peptides from the endosome compartment to the cytosol facilitating cross-presentation in immature DCs (Todryk et al., 1999), which then interact with cytotoxic T cells in an antigen-dependent fashion (Noessner et al., 2002; Binder and Srivastava, 2005). Also, HSPs undergo receptor-mediated endocytosis in DCs (Arnold-Schild et al., 1999), and induce maturation and migration of DCs (Binder et al., 2000). HSP-70 is not normally expressed in the brain under physiological conditions but is highly induced after ischemia (Planas et al., 1997; de la Rosa et al., 2013). HSP-70 is released from necrotic cells to the extracellular space (Todryk et al., 1999), and it can reach the bloodstream (Campisi and Fleshner, 2003). Because of the immunological properties of HSPs, the high induction of HSP-70 in the ischemic brain, and the presence of HSP-70 in the blood, led us to deduce that APCs in peripheral lymphoid tissue might carry HSP-70 after stroke. Indeed, we found that stroke patients showed higher immunoreactivity to HSP-70 and more HSP-70 immunoreactive APCs in lymphoid tissue than the controls, and that stronger presence of HSP-70 in lymphoid tissue was associated with smaller infarctions and better functional outcome (Gómez-Choco et al., 2014). Although a causal relationship between the presence of HSP-70 in the lymphoid tissue and the better outcome of the patients was not proved, it is feasible that the immunoregulatory properties of HSP-70 could modulate autoimmune responses after stroke. In other situations, HSP-70 has been implicated in the development of autoimmunity by promoting inflammatory responses, enhancing DC antigen presentation, and cytotoxic lymphocyte function (Millar et al., 2003) and it is involved in direct chaperoning of antigens into DCs (Todryk et al., 1999). While other HSP proteins, such as HSP-90, facilitate cross-presentation by antigen transfer to the cytosol (Imai et al., 2011), recent data support that HSP-70 impairs it (Kato et al., 2012), thus implying that HSP-70 might favor MHC II presentation. Although exposure of DCs to HSP-70 attenuates T cell responses (Stocki et al., 2012) and HSP-70 improves the immunosuppressive functions of Tregs, it also activates effector T cells (Wachstein et al., 2012). However, brain-derived HSP-70 has been involved in the induction of regulatory NK cells that can induce tolerance in experimental autoimmune encephalomyelitis (Galazka et al., 2006, 2007). Therefore, HSP-70 exerts diverse and complex actions in the immune system and further study is required to understand its role in stroke immunity.

## Antigen-specific T cells

Whether the immune response to acute brain injury is non-specific or is directed against specific brain antigens is not yet settled. While tolerogenic effects have been reported with vaccination using neural antigens, several lines of evidence suggest that T cell accumulation at the site of traumatic CNS injury lacks selectivity, as shown after systemic administration of passively transferred T cells recognizing either neural self-antigen or non-self-antigen, since it resulted in accumulation of the T cells in injured optic nerve regardless of the antigen used for immunization (Hirschberg et al., 1998). The latter effects could be more related to non-specific effects of T cells described at early reperfusion following brain ischemia (Kleinschnitz et al., 2010). Nonetheless, evidences for antigen-specific T-cell reactivity have been found in animal models of acute brain injury. Indeed, nerve trauma can trigger the expansion of myelin-reactive T lymphocytes (Olsson et al., 1992) and an abnormal abundance of T cells autoreactive to myelin was reported in peripheral nerve trauma (Olsson et al., 1992), or spinal cord injury (Kil et al., 1999). While in the spinal cord endogenous MBP-reactive lymphocytes activated by traumatic injury can contribute to tissue damage and impair functional recovery (Jones et al., 2002), several lines of evidence support beneficial effects of these cells in the CNS (Graber and Dhib-Jalbut, 2009). Modulation of immune responses by priming T-cells with neural antigens has shown beneficial neuroprotective and anti-inflammatory actions in models of acute brain injury. Notably, autoreactive type-1 and -2 memory T cells pre-primed with myelin oligodendrocyte glycoprotein (MOG), a protein expressed on the surface of oligodendrocytes and myelin sheaths that is exclusive of nervous system, accelerated re-vascularization and healing following post-traumatic brain injury (Hofstetter et al., 2003). Furthermore, passive transfer of MBP-autoimmune T cells protected injured neurons in the CNS from degeneration (Moalem et al., 1999), and a possible contribution of a neurotrophin-related mechanism was proposed (Barouch and Schwartz, 2002). In experimental stroke, systemic inflammation at the time of MCAO in rats induced the development of a deleterious autoimmune response to MBP after 1 month (Becker et al., 2005). Furthermore, in a similar experimental model, impairment of neurological deficits associated with a Th1 response to MBP in the spleen was reported as soon as 48 h after induction of ischemia (Zierath et al., 2010). Further studies are needed to clarify the time-course development of antigen-specific reactions after stroke and their possible negative impact in functional outcome.

In stroke patients, antigen-specific T-cell reactivity (Tarkowski et al., 1991a,b) and *in vivo* expansion of myelin reactive T cells in the CSF (Wang et al., 1992) were observed more than 20 years ago. Increased influx of MOG-specific T cells into the brain was also detected after experimental stroke (Dirnagl et al., 2007). Th1 responses against MBP ranged from 24% in patients with no stroke-associated infection to 60% in patients with pneumonia and more robust Th1 responses to MBP 90 days after human stroke were associated with a decreased likelihood of good functional outcome, even after adjusting for major independent prognostic factors such as baseline stroke severity and age. Responses to another myelin-associated antigen, myelin proteolipid protein (PLP), and to the astrocyte marker GFAP also seemed to be associated to poor outcome, but reactivity to other antigens such as NSE, S-100β, and tetanus toxin were not predictive of outcome (Becker et al., 2011). The diverse prognostic consequences of immune responses to different brain antigens were also seen when analyzing the presence of brain-derived antigens in the lymphoid tissue of stroke patients. Greater reactivity to MBP was correlated with stroke severity on admission, larger infarctions, and worse outcome at follow-up, whereas increased reactivity to neuronal-derived antigens, such as microtubule-associated protein-2 and NMDA receptor subunit NR-2A, was correlated with smaller infarctions and better long-term outcome (Planas et al., 2012). Interestingly, in cervical lymph nodes of multiple sclerosis patients, neuronal antigens were present in pro-inflammatory APCs, whereas the majority of myelin-containing cells were anti-inflammatory (van Zwam et al., 2009). The authors concluded that the presence of myelin and neuronal antigens in functionally distinct APC populations suggests that differential immune responses can be evoked.

It is not settled whether autoimmune responses are the cause or a consequence of severe ischemic damage but the opposing prognostic implications of immune responses to specific brain antigens do suggest a pathogenic role of autoimmune responses against myelin antigens. Fast-conducting myelinated tracts are responsible for long-range connectivity, interhemispheric synchronization, and also have neurotrophic effects (Dan and Poo, 2004; Nave, 2010) and injury to these fibers can therefore impair brain connectivity (Sun et al., 2011; Lawrence et al., 2013), reduce cortical blood flow, and promote cerebral atrophy (Appelman et al., 2008; Chen et al., 2013). Given that myelination is important for neuroplasticity and motor learning (Fields, 2010), greater autoimmune damage to myelin could also compromise recovery after stroke and contribute to cognitive impairment. It is unknown whether antigen-specific responses to molecules widely expressed in the body might also develop after stroke. Patients with antiphospholipid syndrome are at risk of stroke (Sciascia et al., 2014) and this may have additional implications regarding whether existing autoantibodies can impair the functional outcome of stroke, or whether stroke could exacerbate pre-existing autoimmune responses.

## Induction of immunologic tolerance in stroke

Seminal studies by the team of J. Hallenbeck, K. Becker and colleagues provided evidences supporting that modulating antigen-specific responses could protect the brain in stroke. They found that oral administration of low doses of bovine MBP to Lewis rats prior to transient (3-h) MCAO reduced infarct volume at days 1 and 4 (Becker et al., 1997) demonstrating induced antigen-specific modulation of the immune response. This strategy reduced delayed-type hypersensitivity to MBP and induced-spleen cell proliferation showing that tolerance to this brain antigen was induced. Similar findings were reproduced using MBP for tolerization through nasal instillation in Lewis rats, which showed reduced infarct volume 24 h after 3-h intraluminal MCAO (Becker et al., 2003). Notably, the latter study showed that the protective effect of MBP tolerization could be transferred by administration of splenocytes from MBP-tolerized donors before induction of MCAO, implying that the protective effect of tolerization can be conferred by splenocyte transplantation. In the same line, nasal vaccination with a MOG peptide prior to transient (2-h) MCAO in C57BL/6 mice reduced infarct volume and was more effective than oral MOG tolerization (Frenkel et al., 2003). Besides tolerization with brain-specific antigens, repetitive nasal administration of small doses of E-selectin was also beneficial in experimental stroke. E-selectin is an adhesion molecule involved in leukocyte trafficking to the tissues across the blood vessels and it is strongly induced in the inflamed endothelium. E-selectin expression in the brain vasculature increases after cerebral ischemia (Huang et al., 2000). In prevention studies, nasal instillation of E-selectin potently inhibited the development of ischemic and hemorrhagic strokes in stroke-prone spontaneously hypertensive rats (SP-SHR; Takeda et al., 2002). Moreover, induction of mucosal tolerance to E-selectin through nasal instillation before induction of permanent MCAO (coagulation) in SP-SHR rats improved the outcome by reducing infarct volume at 48 h (Chen et al., 2003). This study also found that adoptive transfer of splenocytes from E-selectin-tolerized donors was able to reduce infarct volume.

But what is the actual mechanism underlying the protection conferred by antigen-specific tolerization against stroke brain damage? Interleukin-10-producing CD4+ T cells mediated the protective effect of nasal tolerization with MOG (Frenkel et al., 2003, 2005), in agreement with the concept that mucosal administration of proteins preferentially induces IL-10 responses mediated by tolerogenic DCs and Tregs (Weiner et al., 2011). Likewise, oral administration of MOG protected against secondary neurodegeneration in a rat model of acute nerve injury by induction of IL-10 producing myelin-reactive T cells (Monsonego et al., 2003). However, nasal MOG tolerization in stroke induced more IL-10 and less CD11b cells than oral MOG (Frenkel et al., 2003). Interleukin-10 production induces unresponsiveness in innate myeloid cells, which then become less capable of generating IL-17-producing encephalitogenic T cells. Oral MBP tolerization induced TGF-β1 and the immunosuppressive features of this cytokine might underlie the beneficial effects of MBP tolerization in stroke (Becker et al., 1997). Mucosal E-selectin tolerization downregulated MHC class I gene expression (Illoh et al., 2006). This effect could prevent the activation of NK cells or cytotoxic T cells and was associated with reduced numbers of CD8+ cells found after ischemia in the tolerized animals (Chen et al., 2003). Tolerization with E-selectin reprogrammed gene expression to inflammation induced by lipopolysaccharide (LPS) promoting the expression of growth factor genes and genes involved in protection against oxidative stress, and it was suggested that E-selectin tolerization could lead to the expansion of Tregs (Illoh et al., 2006). Again, increased expression of IL-10 was also proposed as a mechanism underlying the protective effects of E-selectin tolerization (Yun et al., 2008). Induction of mucosal tolerance triggers Tregs in an antigen-specific fashion (Weiner et al., 2011). Regulatory T cells attenuate inflammation and prevent autoimmunity through secretion of immunosuppressive cytokines, amongst other effects (Costantino et al., 2008). Then, Tregs can exert a global non-specific suppressive effect locally where they encounter the specific antigen, and this action could mediate, at least in part, the beneficial effects of mucosal tolerization in stroke (Frenkel et al., 2005).

## The effect of systemic inflammation

Large community studies have found associations between systemic inflammatory conditions, such as osteorarthritis or pelvic inflammatory disease, and cardiovascular disease, including stroke (Chen et al., 2011; Rahman et al., 2013). This could be the result of the profound implication of many components of the immune system in the pathological processes underlying the development of atherosclerosis and in particular in its ischemic complications (Sherer and Shoenfeld, 2006). Acute ischemic brain damage is also exacerbated by systemic inflammation. In an experimental model of cerebral ischemia, systemic inflammation caused sustained disruption of the tight junction protein, claudin-5, and also exacerbated disruption of the basal lamina collagen-IV, and these alterations were associated with an increase in neutrophil-derived MMP-9 (McColl et al., 2008). In the same line, systemic inflammation in IL-10 deficient mice, spontaneously developing colitis when exposed to environmental pathogens, increased mortality after stroke (Pérez-de Puig et al., 2013). Using a murine model of chronic infection leading to a chronic Th1-polarized immune response, Dénes et al. (2010) found upregulation of proinflammatory mediators in the brain and peripheral tissues, as well as an altered Treg response, accelerated platelet aggregation in brain capillaries, increased microvascular injury and MMP activation after experimental ischemia, and a 60% increase in brain damage.

Infections are the most common complication in stroke patients (Kumar et al., 2010; Westendorp et al., 2011). The most frequent infections are respiratory infections and urinary tract infections and the main clinical predictor of infection is the severity of the neurological deficit. Experimental and clinical studies showed that stroke induces a transient immunodepression that increases the susceptibility to systemic infections in the first days after cerebral ischemia. This was first described in a murine model of cerebral ischemia (Prass et al., 2003) where overactivation of the adrenergic system caused apoptotic loss of lymphocytes and a shift from Th1 to Th2 cytokine production. Atrophy of primary and secondary lymphoid organs and increased numbers of Treg cells were also features of the systemic immune changes induced by cerebral ischemia (Prass et al., 2003; Offner et al., 2006). In stroke patients, the best established features of stroke-induced immunodepression are increased levels of stress hormones and anti-inflammatory cytokines like IL-10 (Haeusler et al., 2008; Klehmet et al., 2009; Urra et al., 2009a), decreased numbers of circulating lymphocytes (Haeusler et al., 2008; Vogelgesang et al., 2008; Urra et al., 2009b), and monocyte deactivation with reduced expression of HLA-DR and reduced capacity to produce inflammatory cytokines (Haeusler et al., 2008; Urra et al., 2009a).

Infection triggers inflammation, facilitates the maturation of APCs into potent immunostimulatory cells (Banchereau et al., 2000), and is involved in the development of autoimmune diseases (Getts et al., 2013; Berer and Krishnamoorthy, 2014). Post-stroke infections complicate the clinical course of the patients (Ulm et al., 2012) and could be a source of inflammation favoring autoimmunity. Systemic inflammation and infection in stroke could set an environment in the periphery favorable to promote the development of effector T cells against brain antigens by providing sufficient cytokines and co-stimulatory molecules (Becker, 2012). However, most features of the stroke-induced systemic immune changes modulate antigen presentation and its consequences, presumably favoring tolerogenic immune responses. Therefore, in the absence of infection, immunodepression would be expected to favor tolerance. Catecholamines can inhibit the antigen-presenting capability via β2-adrenoceptors and this effect is at least partly due to impaired CD8+ cell priming by cross-presenting DC (Seiffert et al., 2002; Maestroni and Mazzola, 2003; Hervé et al., 2013). Corticosteroids inhibit the production of inflammatory cytokines in APCs and induce the development of tolerogenic APCs (DeKruyff et al., 1998; de Jong et al., 1999), and glucocorticoid-stimulated monocytes reduce the release of IFN-γ and IL-17 in lymphocytes favoring the generation of Treg (Varga et al., 2014). Interleukin-10 also inhibits autoimmune reactions acting on several immune cells including APCs. Interleukin-10 treated DCs induce anergic T cells that are able to suppress activation and function of T cells in an antigen-specific manner (Steinbrink et al., 2002). In addition, other alterations in the numbers and phenotype of circulating leukocytes, such as lymphocytopenia and reduced HLA-DR expression in monocytes, could further impair the activation of specific T cell responses against brain antigens. For all these reasons, while predisposing patients to systemic infections, immunodepression after stroke could limit detrimental autoimmune responses in the brain. The effect of infection in stroke has been studied in experimental models of brain ischemia. Induction of ischemia in mice intranasally infected with the human influenza A (H1N1) virus increased the number of neutrophils expressing the MMP-9 in the ischemic brain, exacerbated BBB breakdown, and increased the rate of intracerebral hemorrhages after tissue plasminogen activator treatment (Muhammad et al., 2011). Systemic inflammation at the time of experimental stroke has been used experimentally to mimic the clinical situation of infection to increase the likelihood of developing a detrimental autoimmune response to brain antigens (Becker et al., 2011) by favoring Th1 responses to MBP (Becker et al., 2005; Zierath et al., 2010). However, induction of systemic inflammation or infection in experimental animals at the time of cerebral ischemia would possibly mimic better the clinical scenario of infections precipitating a stroke rather than the infections occurring as a complication of stroke, since the latter are usually related to the severity of the lesion and the degree of stroke-induced immunodepression (Chamorro et al., 2007; Dirnagl et al., 2007). Becker et al. (2011) reported that patients who developed an infection after stroke, especially pneumonia, were more likely to show a Th1 response to MBP and GFAP 90 days after stroke. This is very relevant because stronger Th1 responses to MBP were seen associated to poor functional outcome. However, stroke associated infections are especially frequent in patients with severe strokes (Hug et al., 2009; Urra and Chamorro, 2010) and the development of autoimmune responses could be strongly influenced by the severity of the brain lesion. It is also very likely that the timing of the infections is a key factor in modulating the immune response (Emsley and Hopkins, 2008). Infections before stroke can be a source of inflammation and thrombosis, and can precipitate stroke onset (Elkind et al., 2011). A small clinical study showed that patients with previous infection had greater deficits and increased platelet activation and platelet-leukocyte aggregation compared with patients without infection (Zeller et al., 2005). Thus the presence of previous infections, possibly including subclinical infections, could facilitate the occurrence of stroke and impair functional recovery.

## Pharmacologic regulation of autoreactive T cells after stroke

Reduction of infarct volume after transient ischemia was achieved by immune regulation of myelin-reactive inflammatory T cells using recombinant T cell receptor ligands (RTL), i.e., partial MHC class II molecules covalently bound to myelin peptides acting as partial agonists that deviate autoreactive T cells to become non-pathogenic (Subramanian et al., 2009; Dziennis et al., 2011), again supporting a negative effect of antigen-specific responses in the lesion caused by stroke. Co-inhibitory molecules, like PD-1, regulate the induction and maintenance of peripheral tolerance (Ceeraz et al., 2013). Accordingly, PD-1-deficient mice showed higher inflammatory responses, infarct volume and neurological deficits after brain ischemia (Ren et al., 2011b). However, mice deficient in PD-1 ligands (PD-L) were protected against ischemic brain damage (Bodhankar et al., 2013b), while it has been reported that PD-1 is necessary for Treg-induced protective effects (Li et al., 2014). Therefore, the role of this pathway in the outcome of brain ischemia seems to be quite complex and is not yet fully characterized.

Regulation of the migration of lymphocyte subsets into the CNS can also control autoimmunity. The egress of lymphocytes from lymph nodes requires lymphocytic S1P1 receptors (Matloubian et al., 2004). The main protective mechanism of fingolimod in multiple sclerosis seems to be mediated by internalization of S1P1 that therefore reduces the responsiveness of T cells to the egress signal S1P and favors CCR7-mediated lymphocyte retention in lymph nodes (Pham et al., 2008). Several experimental studies reported protection after treatment with fingolimod in brain ischemia (Hasegawa et al., 2010; Wei et al., 2011; Kraft et al., 2013), and intracerebral hemorrhage (Rolland et al., 2011), but the mechanisms underlying this protection are not fully understood. While lymphocytopenia could be involved in the effects of fingolimod, one study found that this drug was not protective in experimental cerebral ischemia in spite of reducing lymphocyte influx (Liesz et al., 2011). Other effects of this drug, including BBB protection, decreased microvascular thrombosis (Kraft et al., 2013), and reduced hemorrhagic transformation in thromboembolic stroke (Campos et al., 2013) could account, at least in part, for the reported beneficial effects. Whether fingolimod affects autoimmune responses and autoreactive T cell migration after brain ischemia has not been reported and it is unknown whether fingolimod-induced lymphocytopenia might further increase the risk of infection. Interestingly, the drug appears to be safe for the treatment of intracerebral hemorrhage in humans (Fu et al., 2014). In any case, experimental interventions on the immune system in stroke models should pay particular attention to immunodepression and infection as possible causes of neurological impairment and mortality. However, studies identifying post-stroke infection in experimental animals and its possible neurological consequences are difficult and still infrequent (Braun et al., 2007; Engel and Meisel, 2010; Hetze et al., 2013).

Besides systemic infections, factors such as severe arteriosclerosis or other systemic autoimmune diseases are also likely to promote a proinflammatory environment favoring autoimmunity in ischemic stroke. As vascular risk factors and atherosclerosis are common in stroke patients, clinical and experimental studies assessing this possibility and also the potential of commonly used drugs, such as statins, to modulate the immune reactions to stroke would be relevant. Several beneficial effects of statins may be due to immunomodulatory effects, including impaired maturation of DCs with reduced expression of molecules like MHC class II preventing antigen presentation to T cells (Kwak et al., 2000; Yilmaz et al., 2004) and inducing tolerogenic DCs that increase the numbers of Treg cells (Li et al., 2013c). Vaccination is also an attractive approach to induce protective immunity avoiding the progression of atherosclerosis. In experiments in animals, atherosclerosis was reduced by vaccination with oxidized LDL, bacteria containing certain modified phospholipids, or heat-shock protein 65 (Palinski et al., 1995; Maron et al., 2002; Binder et al., 2003).

## Future directions

In this review we described the current evidence suggesting the possibility that stroke can trigger antigen-specific responses. These include the finding of T-cells autoreactive to brain antigens in stroke patients, the presence of brain antigens and autoantibodies in CSF and serum, and APCs carrying brain antigens in the regional lymphoid tissue. Further support to this notion is provided by the beneficial effects of inducing immune tolerance in experimental animal models of stroke. Brain antigens are released after stroke in the presence of inflammatory mediators and danger signals. Soluble molecules can reach the periphery across the leaky BBB or across natural pathways normally allowing fluid efflux, i.e., the drainage of interstitial fluid to the CSF and from there to the blood, and the drainage of CSF through the nasal lymphatics to the cervical lymph nodes. Furthermore, antigens can be internalized locally in the brain by APCs, but whether these cells can migrate to the draining lymph nodes for efficient antigen presentation is currently unknown and deserves further investigation. Since these routes could trigger different immune responses, it is relevant to elucidate their contribution to brain antigen transfer to the lymph nodes. Mechanisms ensuring tolerance to self are tightly regulated. Peripheral tolerance relies on factors including the features of APCs and their interaction with lymphocytes, the cytokine environment, and the presence of danger signals and regulatory or suppressor cells. Although a number of studies have shown specific changes in these factors after stroke, we still lack a complete picture of how these changes are integrated in the organism over time. Furthermore, stroke co-morbidities are often associated with changes in the immune system that could play a crucial role in directing specific immune responses to stroke. Stroke does not consistently trigger autoimmunity, but several lines of evidence support that infection and inflammation could break immune tolerance controls and favor autoreactive responses to brain antigens after stroke. Infection is a frequent complication of stroke that is attributable to stroke-induced immunodepression, characterized by acute lymphopenia and monocyte deactivation. Immunodepression sets a humoral and cellular situation favorable to prevent autoreactivity, but leaves the subjects at risk of infection. In the event of infection, the risk of autoreactivity increases, suggesting a fine balance between the factors regulating tolerance and autoimmunity. Further understanding of these regulatory mechanisms is necessary to elucidate whether antigen-specific reactions could threaten the outcome of stroke patients. Some patients show partial recovery of function and respond to rehabilitation over months after stroke onset. However, certain stroke patients develop complications, as for instance cognitive decline or epilepsy, and it is often difficult to predict such effects. Whether any autoimmune reaction can underlie stroke complications deserves further investigation aiming to prevent or attenuate such adverse events.

## Conflict of interest statement

The authors declare that the research was conducted in the absence of any commercial or financial relationships that could be construed as a potential conflict of interest.
